# High-Performance SnO_2_ Ink for Detecting Low-Temperature H_2_S Gas with Advanced Dispensing Printing Technology

**DOI:** 10.3390/s26196031

**Published:** 2026-09-23

**Authors:** Sufaid Shah, Jesse Nii Okai Amu-Darko, Xingzuo Song, Xiaojin Zhao, Xiaofang Pan

**Affiliations:** 1 College of Electronics and Information Engineering, Shenzhen University, Shenzhen 518060, China; sufaid@szu.edu.cn (S.S.); 2453044005@mails.szu.edu.cn (X.S.); 2 National Key Laboratory of RF Heterogeneous Integration, Shenzhen University, Shenzhen 518060, China; eexjzhao@szu.edu.cn; 3 College of Physics and Optoelectronics Engineering, Shenzhen University, Shenzhen 518060, China; jnodarko@szu.edu.cn; 4 College of Integrated Circuits, Shenzhen University, Shenzhen 518060, China

**Keywords:** dispensing printing, MEMS, gas sensor, tin oxide ink, low-temperature

## Abstract

Hydrogen sulfide (H_2_S) is a highly toxic and environmentally hazardous gas, necessitating sensitive and reliable detection at trace concentrations. In this study, a dispensing-printed SnO_2_-based MEMS gas sensor was developed using a sol–gel-derived SnO_2_ precursor ink deposited directly onto a Pt-interdigitated micro-hotplate. The precursor ink was optimized for dispensing printing and subsequently annealed at 450 °C to produce a crystalline SnO_2_ sensing layer. XRD and FTIR analyses confirmed the formation of crystalline tetragonal rutile SnO_2_ and characteristic Sn–O bonding, while FE-SEM revealed an interconnected particulate sensing morphology with well-distributed Sn and O. XPS further confirmed the predominant Sn^4+^ chemical state and the presence of lattice and surface-related oxygen species, which are beneficial for gas adsorption and surface reactions. The fabricated sensor demonstrated effective H_2_S sensing performance at a relatively low operating temperature of 90 °C. The response increased systematically with the H_2_S concentration from 10 to 100 ppb, reaching a maximum response of 3.8897 at 100 ppb. A sensitivity of 0.0282 response units per ppb was obtained from the calibration curve, demonstrating a strong concentration-dependent response in the investigated range. Additionally, the power consumption of the sensor was low (~32 mW); the sensor also exhibited a response time of approximately 153 s, although recovery was comparatively slower at approximately 277 s at 30 ppb. Furthermore, the sensor showed a substantially higher response to H_2_S than the investigated interfering gases, including H_2_, NH_3_, NO_2_, and CO. These results demonstrate that the integration of dispensing-printed SnO_2_ with a MEMS micro-hotplate provides a promising route toward low-temperature, miniaturized, and highly sensitive trace-level H_2_S sensing.

## 1. Introduction

Hydrogen sulfide (H_2_S) is a toxic and corrosive gas present in petroleum refining, wastewater treatment, mining, agricultural, and various chemical production operations [1,2]. It is a colorless, flammable gas characterized by its distinctive rotten egg odor at low concentrations. Although its strong odor is detectable at concentrations as low as 0.5–1 ppm, olfactory fatigue occurs quickly, making smell an unreliable indicator of exposure [3,4]. Regulatory agencies impose strict limits to ensure workplace safety: the Occupational Safety and Health Administration (OSHA) sets a permissible exposure limit of 20 ppm, while concentrations above 100 ppm can cause immediate health impairment, and levels exceeding 300 ppm may be fatal [5,6,7]. These hazards highlight the urgent need for fast, accurate, and reliable H2S detection, particularly at low temperature and in simple fabrication, low-power systems.

Semiconducting metal oxide (MOS)-based gas sensors have been extensively investigated because of their high sensitivity and response, exceptional chemical stability, low fabrication cost, and compatibility with miniaturized sensing platforms. Various MOS materials, including SnO_2_ [8,9], ZnO [10,11], In_2_O_3_ [12,13], WO_3_ [14,15], TiO_2_ [16,17], CuO [18,19], and NiO [11,20], have demonstrated promising sensing characteristics toward toxic and combustible gases. Among them, tin dioxide (SnO_2_), an n-type semiconductor with a wide bandgap of approximately 3.6 eV [21], has attracted particular attention owing to its exceptional electrical conductivity, thermal stability, chemical durability, and strong affinity for sulfur-containing gases [22,23,24]. The adsorption of H_2_S molecules on the SnO_2_ surface induces significant changes in electrical resistance, enabling highly sensitive detection. Moreover, SnO_2_ can be synthesized in diverse nanostructured forms, including nanoparticles, nanowires [25], nanosheets [26], nanotubes [27], and porous architectures [28], which provide abundant active sites and enhanced gas diffusion for improved sensing performance. Despite these advantages, conventional SnO_2_ gas sensors generally require elevated operating temperatures between 200 and 450 °C to activate surface oxygen species responsible for gas sensing reactions [28,29]. Such high working temperatures increase power consumption, reduce device lifespan, and limit integration into portable, wearable, and battery-powered sensing systems. Significant efforts have been devoted to producing low-temperature SnO_2_ gas sensors that combine great sensitivity with ultralow detection limits.

Inkjet and dispensing printing technologies have received significant attention for gas sensor fabrication due to their ability to deposit diverse functional materials, including nanoparticles, polymers, semiconductors, and metal oxides, with high spatial precision. Recent advances in printed electronics have created new opportunities for manufacturing flexible, lightweight, and cost-effective gas sensors [30,31,32]. Compared with conventional fabrication techniques, including chemical vapor deposition, physical vapor deposition, and photolithography, printing technologies offer scalable production, reduced material waste, simplified processing, and compatibility with diverse substrates. Among these approaches, dispensing printing technology enables precise deposition of functional inks with high pattern fidelity and thickness control, making it particularly attractive for fabricating miniaturized sensing devices and microelectromechanical system (MEMS)-based sensor arrays. Previous studies have produced SnO_2_ inks printed on various substrates, showing promising sensitivity after annealing and improved response characteristics [33,34,35].

In this work, an advanced SnO_2_ ink was synthesized through a modified sol–gel route to produce highly dispersed precursor nanoparticles with diameters below 100 nm. The optimized ink exhibited colloidal stability and rheological properties suitable for dispensing printing technology, enabling uniform deposition onto MEMS sensor platforms. Following low-temperature annealing below 500 °C, highly crystalline SnO_2_ sensing films were obtained with well-defined structural integrity. The fabricated printed sensor demonstrated low-temperature H_2_S sensing performance at an operating temperature of 90 °C, including high sensitivity, fast response and recovery, good repeatability, and stable long-term operation. The novelty of the present work lies in achieving low-temperature operation for an undoped SnO_2_, yielding sub-ppm detection via high dispersion compatible with the proposed MEMS platform. This printing strategy provides a scalable and cost-effective route for manufacturing next-generation low-power H_2_S gas sensors suitable for industrial safety monitoring, environmental protection, and portable smart sensing systems.

## 2. Materials and Methods

### 2.1. Reagents and Materials

A MEMS-based micro-hotplate (MHP) sensor chip (SA-MHP-3.8, Beijing, China) with an overall size of 3.8 × 3.8 mm^2^ and a central heating area of 0.3 × 0.3 mm^2^ was used as the sensing substrate. The chip consisted of a suspended membrane integrated with Pt interdigital electrodes and was capable of operating at temperatures up to 550 °C. Tin chloride dihydrate (SnCl_2_·2H_2_O, Alfa Aesar, Tianjin, China) was used as the Sn precursor, while ethanolamine (J&K, Shanghai, China) served as the coordinating solvent. Isopropanol (IPA) and ethanol were purchased from Nanjing Chemical Reagent Co. (Nanjing, China). Ammonia solution (NH_3_·H_2_O, 28%, *v*/*v*) was obtained from Shanghai Lingfeng Chemical Reagent Co. (Shanghai, China), and nitric acid (HNO_3_) was purchased from Merck (Zurich, Switzerland). Ultrapure water (18.2 MΩ·cm) was produced using a Milli-Q purification system (Millipore, Bedford, MA, USA). Unless otherwise specified, all reagents were of analytical grade and used as received.

### 2.2. Preparation and Optimization of SnO_2_ Ink

The SnO_2_ ink was prepared by a sol–gel route using SnCl_2_·2H_2_O as the tin precursor. Briefly, 0.85 g of SnCl_2_·2H_2_O was dissolved in 10 mL of pure ethanolamine, which acted as a bidentate ligand to stabilize the tin ions in solution. The mixture was ultrasonicated for 30 min to yield a transparent tin complex solution, then heated and stirred at 70 °C for 2 h to obtain a homogeneous, light-yellow precursor sol. After cooling to room temperature, 1 mL of the precursor sol was combined with 0.550 mL of concentrated acetic acid, added slowly via syringe pump to maintain a stable pH and control the resulting particle size and morphology, and the mixture was stirred for a further 2 h to develop the ink rheological properties. The final ink exhibited a viscosity of 10–12 cP and a surface tension of 25–40 mNm^−1^, suitable for consistent droplet formation and clog-free dispensing from the printer’s 60 μL syringe. A portion of the ink was separately sintered in an oxygen-rich atmosphere at 450 °C for 10 h to yield powdered nano-SnO_2_ for reference characterization of the crystalline phase and particle size.

### 2.3. MEMS Substrate Preparation and SnO_2_ Sensor Fabrication

Prior to printing, the MEMS chips were sequentially cleaned by immersion in acetone, isopropanol, and deionized water to remove organic and particulate contaminants. The cleaned substrates were dried at 120 °C for 1 h. Where required, an oxygen-plasma treatment was applied using a CCI-PLA100 plasma reactor (Scientific 3, Shanghai Zhongmei Technology Co., Ltd., Shanghai, China) at 100 W and 40 kHz for 2 min to modify the surface energy and improve the adhesion of the SnO_2_ ink. The optimized SnO_2_ ink was then dispensing-printed directly onto the Pt interdigital electrodes of the MEMS chip. Printing parameters, including dispensing speed (DS: 2 mm/s), dispensing pressure (DP: 3 kPa), nozzle-to-substrate distance (DH: 31.94 mm), and the thickness of film (2.412 μm) were optimized to obtain stable droplet formation and uniform coverage of the active sensing region while avoiding deposition on nonfunctional areas. The printed films were initially dried at 50 °C and subsequently annealed at 450 °C for 10 h. The thermal treatment promoted crystallization of SnO_2_ and removed residual organic components from the precursor, yielding the final SnO_2_-based MEMS gas sensor. A schematic representation of the overall ink preparation, MEMS fabrication, and gas sensing process is presented in Figure 1.

### 2.4. Gas Sensing Measurements

Gas sensing measurements were conducted using a CGS-8 intelligent gas sensing analysis system (Beijing Elite Tech Co., Ltd., Beijing, China; Figure 2a,b. Following the preparation and optimization of the SnO_2_ ink, the MEMS substrate was characterized and subsequently functionalized with the sensing material; Figure 2c,d summarize the electrode configuration based on SA-MHP-3.8 MEMS micro-hotplate with Pt heater and Pt sensing electrodes. The heater resistance was 150 Ω, with the final deposition of the SnO_2_ layer on the active MEMS sensing region. (H_2_S) was selected as the target gas to evaluate the sensing performance of the fabricated SnO_2_ MEMS sensor. A certified H_2_S standard gas with a concentration of 1.00 × 10^−6^ mol mol^−1^ (1 ppm) and a relative expanded uncertainty of 2% (k = 2) was used as the source gas. The certified standard was prepared and characterized in accordance GB/T 5274.1-2018, Gas Analysis—Preparation of Calibration Gas Mixtures—Part 1: Gravimetric Method for Class I Mixtures [36]. Standardization Administration of China: Beijing, China, 2018. while the relevant measuring instruments were verified/calibrated by Dalian Metrology Inspection and Testing Research Institute Co., Ltd. (Dalian, China). The required H_2_S concentrations were obtained by controlled dilution of the certified standard gas with carrier gas using the gas sensing test system at approximately 27 °C and 13% RH with a total gas flow rate of 500 mL/min. The sensing resistance was continuously recorded using the built-in measurement circuitry of the CGS–8 system without an additional external bias source. A single independently fabricated SnO_2_ MEMS device was used for the gas sensing experiments. The resistance was continuously recorded using the CGS–8 gas sensing system with 1 s acquisition intervals. To investigate the selectivity of the sensor, hydrogen (H_2_), ammonia (NH_3_), nitrogen dioxide (NO_2_), and carbon monoxide (CO) were employed as potential interfering gases under identical experimental conditions. The test gases were introduced individually into the sensing chamber at predetermined concentrations using calibrated gas-flow control, including a 100 mL chamber equipped with temperature monitoring, and the corresponding resistance changes were recorded. All gas concentrations and flow rates were controlled consistently during the sensing experiments to enable reliable comparison of the sensor response toward H_2_S and the interfering gases. The MEMS micro-hotplate was used to control the operating temperature through the integrated heater and calibrated using the temperature–current relationship obtained from infrared thermal imaging. The sensor repeatability measurements, response, response/recovery characteristics, concentration dependence, and temperature-dependent sensing behavior were systematically investigated. Gas responses were determined using the equations R = Rg/Ra for oxidizing gases and R = Ra/Rg for reducing gases, where Rg and Ra represent resistance in the target and air, respectively.

## 3. Results and Discussion

### 3.1. Structural and Morphological Characterization

X-ray diffraction (XRD) was utilized to determine the crystalline structure of the prepared SnO_2_ ink formed after the heat treatment process. This was conducted with a Philips Xpert PRO diffractometer. The XRD patterns were recorded over a 2θ range of 5° to 80°, with a step width of 0.02°, utilizing Cu-Kα radiation (λ = 1.5406 Å). The XRD results, as shown in Figure 3, clearly identified the crystalline phases present in the SnO_2_, with prominent diffraction peaks corresponding to the (110), (101), (200), (211), and other planes, which are consistent with the JCPDS/PDF card# 41-1445 database for SnO_2_ [37] (Figure 3a). Among the observed reflections, the (110) reflection was the most prominent, which is characteristic of the rutile SnO_2_ phase. The presence of multiple well-resolved reflections, together with the absence of additional intense peaks attributable to secondary crystalline phases, indicated that the thermal treatment effectively produced the desired SnO_2_ phase without detectable crystalline impurities [38]. This observation is particularly important for the dispensing-printed sensor because residual precursor-derived phases or incomplete conversion could alter the electrical conductivity and surface chemistry of the sensing layer. The XRD patterns confirmed the formation of a tetragonal rutile structure characteristic of SnO_2_, with prominent peaks corresponding to the (110), (101), (200) and (211) planes. The sharpness and intensity of these peaks indicated the formation of a well-crystallized tetragonal SnO_2_ phase, which is essential for efficient gas sensing.

The chemical bonding characteristics of the annealed SnO_2_ material were further examined by FTIR spectroscopy, as shown in Figure 3b. The spectrum exhibits characteristic absorption features associated primarily with Sn–O/Sn–O–Sn vibrations in the low-wavenumber region. The bands observed approximately within 460–600 cm^−1^ can be assigned to metal–oxygen vibrational modes associated with Sn–O and Sn–O–Sn bonds, providing further evidence of the formation of the SnO_2_ oxide network [39]. In addition to the Sn–O-related vibrations, a broad absorption band in the ~3350–3600 cm^−1^ region was observed, which as attributed mainly to the stretching vibration of hydroxyl (–OH) groups. A weaker band around 1623 cm^−1^ was associated predominantly with the bending vibration of O–H bonds, generally related to physically adsorbed moisture or surface hydroxyl species [40,41]. These surface hydroxyl groups can originate from atmospheric moisture adsorbed onto the high-surface-area SnO_2_ material during handling or FTIR measurement. The presence of these weak hydroxyl-related features does not indicate incomplete formation of SnO_2_. Rather, surface hydroxylation and adsorbed water are common for metal oxide surfaces and can play an important role in gas sensing reactions by influencing surface charge, oxygen adsorption, and interaction with analyte molecules. In particular, the surface hydroxyl species may contribute to the adsorption and reaction processes occurring at the SnO_2_–gas interface during sensing. Notably, the FTIR spectrum does not show prominent absorption bands associated with the organic precursor components, indicating that the heat treatment effectively removed the major organic constituents of the ink. This observation is consistent with the XRD results, which demonstrate the formation of crystalline SnO_2_. The combined XRD and FTIR analyses therefore confirm that the thermal treatment successfully converted the printed precursor into a predominantly crystalline SnO_2_ oxide layer while eliminating substantial residual organic species.

To further elucidate the surface chemical composition and oxidation states of the dispensing-printgedSnO_2_ sensing layer, X-ray photoelectron spectroscopy (XPS) analysis was performed. The corresponding XPS spectra are presented in Figure 4a–c. The survey spectrum in Figure 4a is dominated by signals associated with Sn and O, confirming that these were the principal elements present at the surface of the annealed sensing layer. No pronounced additional elemental signals were observed, supporting the high chemical purity of the SnO_2_ material and the effective removal of the precursor-derived species during the annealing process. The high-resolution Sn spectrum in Figure 4b exhibits two distinct peaks centered at approximately 486.81 and 495.26 eV, corresponding to the Sn 3d5/2 and Sn 3d3/2 components, respectively. The separation between the two components is characteristic of the spin–orbit splitting of Sn 3d states. The observation of the Sn 3d doublet at these binding energies is consistent with the presence of Sn^4+^ in SnO_2_, confirming the successful formation of the expected tin oxide chemical state. The absence of an obvious additional lower-binding-energy Sn component suggests that reduced tin species, such as Sn^2+^, were not significant at the surface within the detection capability of the measurement [42]. The O 1s spectrum was further deconvoluted to provide insight into the different oxygen environments present on the SnO_2_ surface, as shown in Figure 4c. Two principal components were identified at approximately 530.38 and 531.58 eV. The lower-binding-energy component at 530.38 eV (Olat) is assigned to lattice oxygen associated with Sn–O bonds in the SnO_2_ crystal structure [43]. The higher-binding-energy component centered at approximately 531.58 eV (Oads) is associated with surface-adsorbed oxygen species and/or oxygen-containing surface species [44]. Such species are particularly relevant to the gas-sensing behavior of SnO_2_ because chemisorbed oxygen can extract electrons from the semiconductor surface and generate an electron-depletion region. Upon exposure to a reducing gas such as H_2_S, these reactive surface oxygen species can participate in surface oxidation reactions, releasing electrons back to the SnO_2_ conduction band and consequently modulating the sensor resistance.

Following confirmation of the crystalline SnO_2_ phase, the morphology and elemental composition of the printed sensing layer were further investigated using field-emission scanning electron microscopy (FE-SEM) and energy-dispersive X-ray spectroscopy (EDS). FE-SEM characterization was performed using a ZEISS Gemini SEM 560 (ZEISS, Oberkochen, Germany) with a resolution of 0.5 nm at 15 kV. As shown in Figure 5a, the low-magnification FE-SEM image reveals the morphology of the SnO_2_ sensing layer deposited over the active MEMS sensing region. The underlying MEMS architecture, including the electrode pattern and suspended-membrane features, remains distinguishable, while the SnO_2_ material is distributed across the sensing area. At higher magnification, as shown in Figure 5b, the deposited layer appears as an interconnected network of irregular, micrometer- and sub-micrometer-scale material with representative lateral dimensions of approximately 2.2–3.1 µm features separated by interparticle boundaries and surface voids. Rather than forming a completely continuous dense film, the particles assembled into interconnected clusters, generating numerous interparticle boundaries and surface voids. Such a cluster architecture is advantageous for gas sensing because it increases the accessible surface area and provides pathways for gas molecules to diffuse into the sensing layer. The relatively rough and porous morphology observed after annealing was attributed to the conversion and decomposition of the precursor ink during the thermal treatment. Removal of volatile and organic components during annealing promotes the formation of interconnected oxide particles and prevents the sensing layer from becoming excessively compact. The resulting interconnected structure is particularly important for the MEMS sensor because the sensing reaction occurs predominantly at the SnO_2_ surface and at regions where gas molecules can readily access the active material.

The elemental composition of the deposited layer was examined by EDS, as shown in Figure 5c. The spectrum is dominated by signals corresponding to Sn and O, confirming the presence of the expected constituent elements of SnO_2_. The corresponding elemental mapping shown in the inset of Figure 5c further demonstrates the spatial distribution of Sn and O throughout the selected sensing area. The broadly overlapping distribution of the two elements is consistent with the formation of a continuous Sn–O-based sensing material rather than isolated regions containing only one of the constituent elements. The combination of the morphological and elemental analyses therefore confirms the successful deposition of SnO_2_ onto the MEMS sensing platform.

### 3.2. Gas Sensing Performance

The gas sensing characteristics of the fabricated sensor toward various target gases at different operating temperatures were systematically investigated to determine its optimum sensing performance. As shown in Figure 6a, the sensor exhibits a pronounced dependence of response on operating temperature. The response initially increases with increasing temperature, reaches a maximum at 90 °C, and subsequently decreases at higher temperatures. This behavior is typical of semiconductor metal oxide gas sensors and reflects the dynamic balance between gas adsorption, surface reaction kinetics, and desorption processes [45]. At relatively low temperatures, the adsorption of oxygen molecules and H_2_S molecules is favorable; however, insufficient thermal energy limits the surface reaction rate, resulting in relatively low sensor response. As the operating temperature increases, the activation energy required for charge transfer reactions is overcome, facilitating the formation of highly reactive chemisorbed oxygen species (O_2_^−^, O^−^), thereby enhancing electron exchange between the sensing material and adsorbed gas molecules [46]. Consequently, the sensor response reaches its maximum at 90 °C, indicating that this temperature provides the optimum balance between adsorption and reaction kinetics. Beyond this temperature, accelerated desorption of gas molecules shortens their residence time on the sensor surface, reducing the number of effective reaction sites and leading to a gradual decline in response.

The relatively low optimum operating temperature demonstrates the improved catalytic activity and abundant active adsorption sites of the synthesized sensing material, which are advantageous for reducing power consumption and improving practical applicability compared with conventional metal oxide gas sensors operating above 200 °C. The dynamic sensing transients of the sensor toward different H_2_S concentrations recorded at the optimum operating temperature of 90 °C are presented in Figure 6b. Upon the introduction of H_2_S into the sensing chamber, the sensor resistance decreased promptly, confirming the typical behavior of an n-type semiconductor exposed to a reducing gas. When the gas flow was terminated and replaced with dry air, the resistance gradually recovered to its initial baseline, demonstrating the reversible nature of the sensing process.

The sensor exhibited stable and repeatable responses over the investigated concentration range of 10–100 ppb, with the response magnitude increasing proportionally with increasing H_2_S concentration. The highest response reached 3.8897 at 100 ppb, indicating high sensitivity toward trace-level H_2_S detection. Furthermore, each sensing cycle showed negligible baseline drift and stable reproducibility, suggesting that the sensing material maintains stable adsorption–desorption characteristics during repeated exposure. The calibration plot of sensor response as a function of H_2_S concentration is shown in Figure 6c. A nearly linear increase in response is observed throughout the investigated concentration range, indicating exceptional quantitative detection capability. The linear fitting yields a sensitivity indicating that the sensor response increases by approximately 0.0282 for every 1 ppb increase in H_2_S concentration within the measured range. The data were well-described by the linear regression y = 0.089973 + 0.0282x (R^2^ = 0.97728). The linearity suggests that adsorption sites remain unsaturated and that electron transfer is proportional to analyte concentration under these operating conditions. The sensor exhibited a strong linear concentration–response relationship over the tested 10–100 concentration-unit range, with R^2^ = 0.9773, indicating good sensitivity within this interval. Based on the calibration residual standard deviation and slope, the estimated detection limit $\left(LOD=\frac{3\sigma }{s}\right),\;$ where σ represents the standard deviation of the blank/baseline response, and s is the calibration sensitivity, which was approximately 3.498 ppb. Such linear behavior is highly desirable for practical environmental monitoring and quantitative gas analysis. To verify gas sensor repeatability, the SnO_2_ sensor was exposed to 30 ppb H_2_S over four cycles, as depicted in Figure 6d. The peak (post-recovery) resistance values recorded after each cycle showed a slight, near-linear upward drift over the course of the measurement, characterized by a regression value of R = 1.2456 ± 0.02817. This modest drift is attributable to the incomplete recovery of the surface oxygen coverage between consecutive H_2_S exposure cycles at 90 °C, a common feature of chemiresistive oxide sensors operated substantially below the temperature required for complete thermal desorption of reaction by-products.

Representative transient curves used to determine the response and recovery times are shown in Figure 7a,b. The response time, defined as the time required to reach 90% of the total resistance change after gas introduction, was approximately 134 s and 153 s at 100 ppb and 30 ppb respectively, whereas the recovery time, defined as the time required for the resistance to return to 90% of its original value after removal of H_2_S, was approximately 283 s and 277 s at 100 ppb and 30 ppb respectively. These were determined directly from the recorded sensing cycles with no additional data processing applied. The fast response indicates efficient diffusion of H_2_S molecules through the porous sensing layer and their immediate interaction with surface oxygen species. Conversely, the relatively longer recovery time can be attributed to the gradual desorption of sulfur-containing intermediate species and the slower readsorption of oxygen molecules onto the sensing surface [47,48]. Nevertheless, the obtained response time compares favorably with that of many reported low-temperature H_2_S sensors and confirms the increased accessibility of the active sites within the sensing architecture. The selectivity of the sensor toward various interfering gases is summarized in Figure 7c. Among all tested gases, the fabricated sensor exhibited a remarkably stronger response toward H_2_S than toward other analytes, confirming its high selectivity. The superior selectivity can be attributed to the strong chemical affinity between H_2_S molecules and the metal oxide surface [49]. H_2_S possesses relatively low bond dissociation energy and readily reacts with chemisorbed oxygen species, producing SO_2_ and H_2_O while releasing electrons back into the conduction band [50,51]. This reaction results in a substantially larger modulation of the depletion layer than that produced by competing gases. Moreover, the favorable adsorption energy and faster surface reaction kinetics of H_2_S contribute to its preferential interaction with the sensing surface, thereby suppressing cross-sensitivity toward other gases. Such high selectivity is particularly advantageous for practical environmental monitoring and industrial safety applications where complex gas mixtures are commonly encountered. For comparison with previously reported H_2_S gas sensors, we achieved smaller volume and low power consumption (PC~32 mW at 90 °C) with different sensing performance, as the proposed SnO_2_ sensor is summarized alongside representative sensors reported in the literature in Table 1. Additionally, repeated exposure to 100 ppb H_2_S gas did not significantly affect the sensing materials responses, suggesting their long-term stability Figure 7d. Over a 30-day repeated operation period, the sensor retained ~85.7% of its initial response, corresponding to a ~14.3% change. This level of drift (~0.5% per day) is consistent with typical aging behavior of nanostructured metal oxide sensing layers, where gradual surface reconstruction and adsorption of ambient species reduce the density of active sites. The observed stability suggests that the present formulation provides a reasonable compromise between sensitivity and durability for short- to medium-term deployments. This gradual decline is typical of chemiresistive metal oxide sensors and is generally attributed to slow, irreversible surface reconstruction, adventitious contamination, or partial loss of surface-active sites over repeated thermal cycling and gas exposure [52].

### 3.3. Gas Sensing Mechanism

The sensing behavior of the dispensing-printed SnO_2_ MEMS sensor toward H_2_S can be primarily understood from the surface-oxygen-mediated charge-transfer process and the modulation of the electron-depletion layer and interparticle potential barriers. The interconnected SnO_2_ particles observed by FE-SEM provide a large number of accessible surface sites and grain/grain-contact regions, which are important for the adsorption of oxygen and subsequent interaction with H_2_S. When the SnO_2_ sensor is exposed to air, oxygen molecules are preferentially adsorbed on the SnO_2_ surface and capture electrons from the conduction band of the n-type semiconductor [28,52]. Depending on the operating temperature, adsorbed oxygen can exist predominantly as ionic species such as O_2_^−^ and O^−^:

O_2_ + e^−^ → O_2_^−^ (ads) (1)

O_2_^−^ (ads) + e^−^ →2O^−^ (ads) (2)

The extraction of conduction-band electrons generates an electron-depletion layer (EDL) near the SnO_2_ surface, thereby increasing the resistance of the sensing layer. At the same time, oxygen adsorption at the interfaces between neighboring SnO_2_ particles produces potential barriers that impede electron transport across the interparticle contacts. Consequently, both the increased depletion-layer width and interparticle potential barriers contribute to the relatively high resistance of the sensor in air [58].

When H_2_S is introduced, it interacts with the chemisorbed oxygen species on the SnO_2_ surface. Because H_2_S is a reducing gas, its oxidation by surface oxygen releases the electrons previously trapped by the adsorbed oxygen species back into the SnO_2_ conduction band. The overall surface reaction can be represented as (Figure 8a):

H_2_S (ads) + 3O^−^ (ads) → SO_2_ + H_2_O + 3e^−^ (3)

The returned electrons reduce the width of the electron-depletion layer and consequently lower the resistance of the n-type SnO_2_ sensing layer. Simultaneously, the reduction in surface charge decreases the potential barrier at the SnO_2_/SnO_2_ particle boundaries, facilitating electron transport through the interconnected sensing network, as seen in Figure 8b. These coupled effects produce the pronounced resistance decrease observed in the dynamic H_2_S response curves.

The relatively low optimum operating temperature of 90 °C can also be understood from the balance between adsorption and surface reaction kinetics. At temperatures below the optimum, thermal energy may be insufficient to promote quick reaction between H_2_S and chemisorbed oxygen species, limiting the rate of charge transfer [59]. Increasing the temperature to 90 °C enhances the reaction kinetics and enables efficient interaction between H_2_S and surface oxygen, resulting in the maximum response of 3.8897 at 100 ppb H_2_S. At excessively high temperatures, however, accelerated desorption of physically and chemically adsorbed gas species can reduce the population of reactive surface oxygen and shorten the residence time of H_2_S molecules, thereby limiting the response. Therefore, the substantial H_2_S response at only 90 °C can be attributed to the combined effects of surface oxygen adsorption, electron-depletion-layer modulation, interparticle potential barrier modulation, and efficient gas accessibility within the printed SnO_2_ network. This mechanism is proposed by analogy with established SnO_2_- and H_2_S-sensing models rather than as a directly verified reaction pathway.

## 4. Conclusions

In this work, a dispensing-printed SnO_2_-based MEMS gas sensor was developed for low-temperature detection of trace H_2_S. A printable SnO_2_ precursor ink was prepared through a sol–gel route and optimized to achieve suitable rheological properties for direct deposition onto a MEMS micro-hotplate. The printed sensing layer was subsequently annealed at 450 °C, resulting in the formation of crystalline SnO_2_. The fabricated sensor exhibited high H_2_S sensing performance at a relatively low operating temperature of 90 °C. The response increased with increasing H_2_S concentration and reached a maximum response of 3.8897 at 100 ppb H_2_S, with a calculated sensitivity of 0.0282 response units ppb within the investigated concentration range. A fast response time of approximately 153 s was achieved, although the recovery process was comparatively slower, with a recovery time of approximately 277 s at 30 ppb. The sensor also demonstrated a substantially stronger response toward H_2_S than the investigated interfering gases, including H_2_, NH_3_, NO_2_, and CO, indicating good selectivity. Overall, the integration of a printable SnO_2_ sensing material with a MEMS micro-hotplate provides a promising strategy for miniaturized, low-temperature, and trace-level H_2_S detection, demonstrating the potential of inkjet printing for scalable fabrication of MEMS-based metal oxide gas sensors.

## Figures and Tables

**Figure 1:**
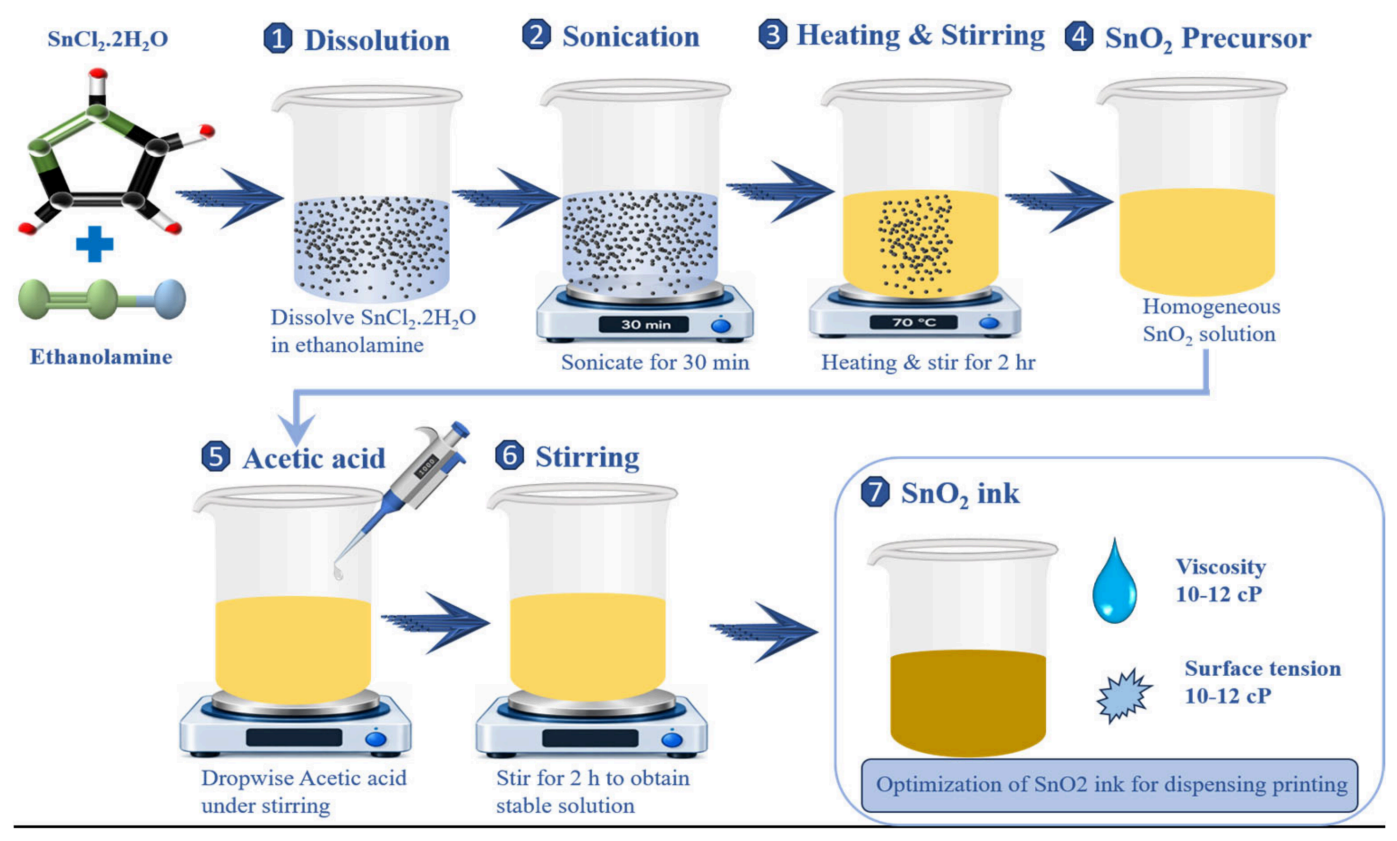
Scheme of preparation of SnO_2_ ink.

**Figure 2:**
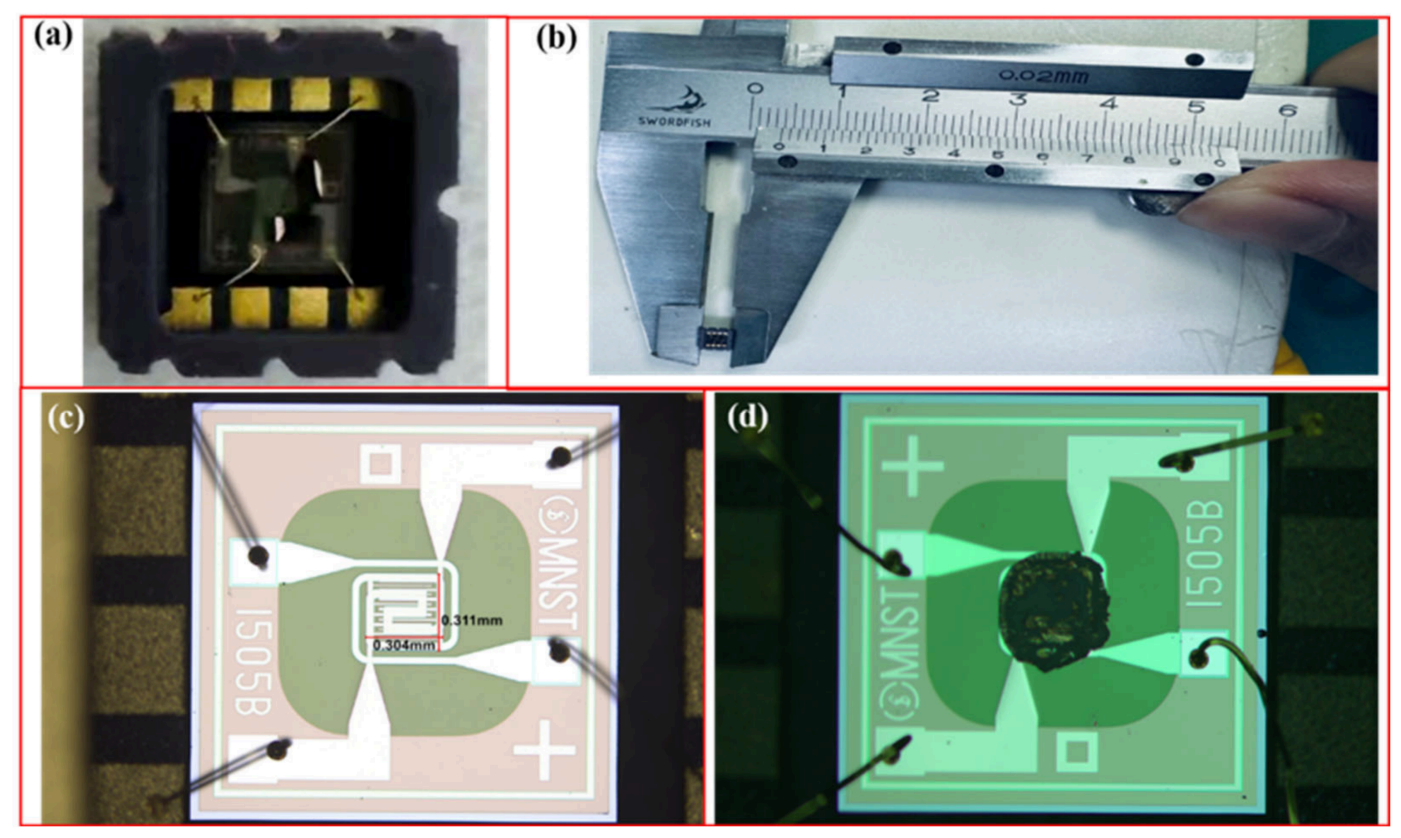
(**a**) Photographs of the topside (electrodes) of the fabricated SnO_2_-coated MEMS sensor device; (**b**) the dimensions of the fabricated sensor structure. (**c**) Microscopic image of the bare MEMS sensing platform showing the Pt interdigital electrode configuration and characteristic dimensions of the central sensing region; (**d**) corresponding microscopic image of the MEMS device after deposition of the SnO_2_ sensing material onto the active sensing area.

**Figure 3:**
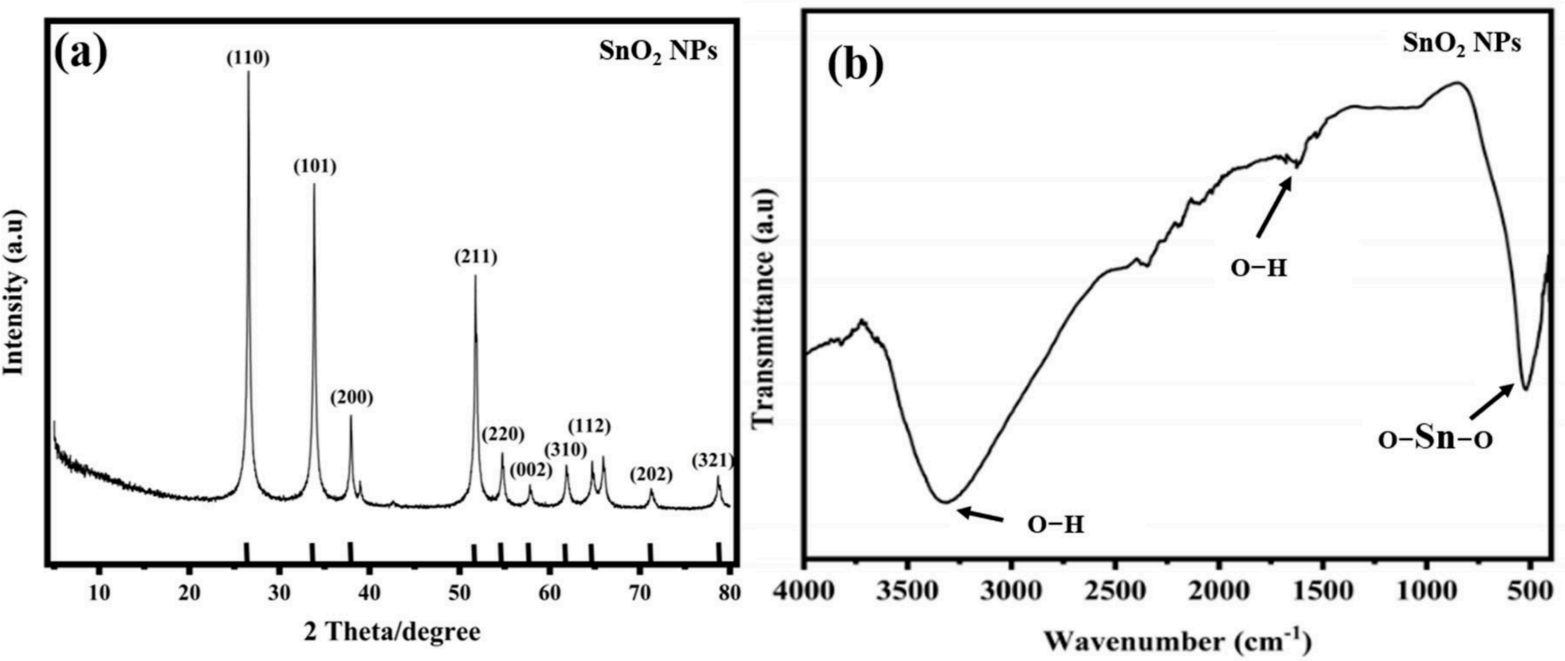
(**a**) XRD pattern of SnO_2_ NPs showing characteristic diffraction reflections of tetragonal rutile SnO_2_; (**b**) FTIR spectrum showing characteristic Sn–O/Sn–O–Sn vibrational bands together with surface hydroxyl/water-related absorption features.

**Figure 4:**
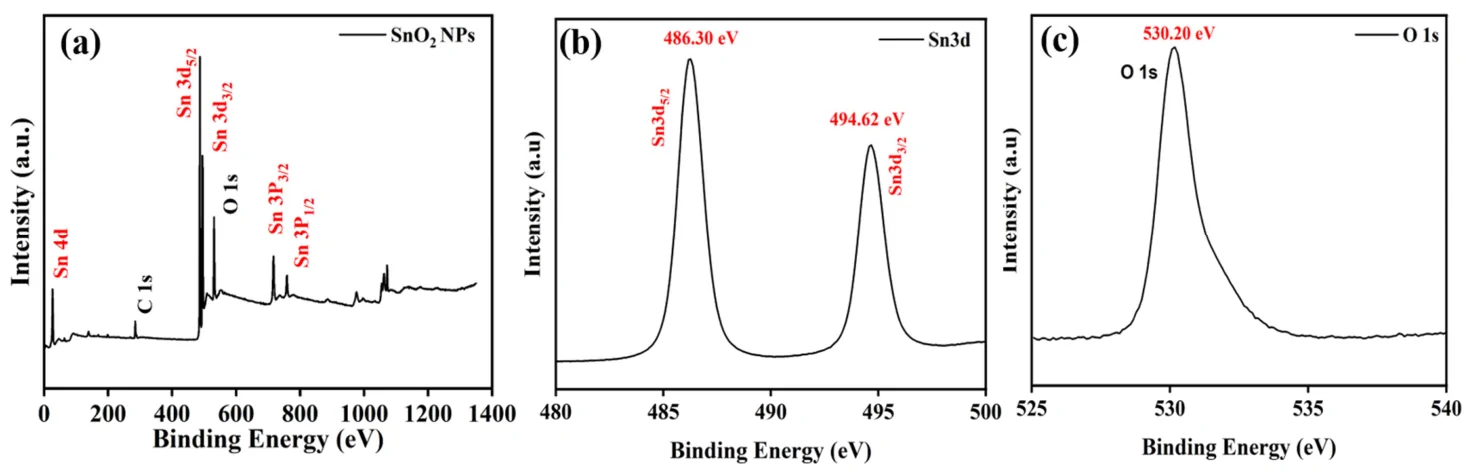
XPS characterization of annealed SnO_2_ sensing layer: (**a**) wide-scan XPS spectrum showing principal Sn and O signals; (**b**) high-resolution Sn 3d spectrum; (**c**) deconvoluted O 1s spectrum showing lattice oxygen and surface-related/adsorbed oxygen species.

**Figure 5:**
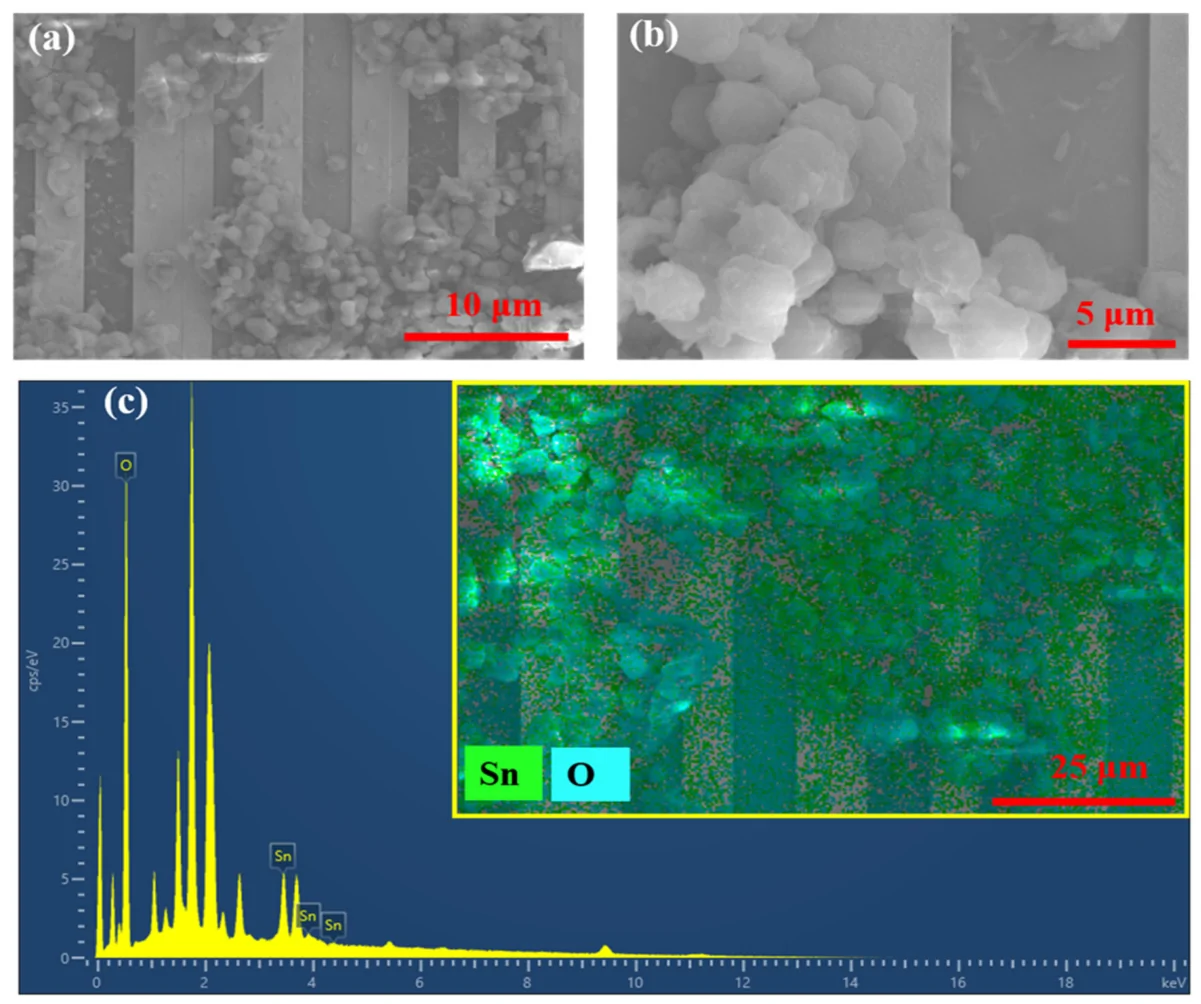
(**a**) Low-magnification FE-SEM image showing the distribution of the SnO_2_ layer over the MEMS sensing region; (**b**) high-magnification FE-SEM image revealing the interconnected particulate morphology of the annealed SnO_2_ layer; (**c**) EDS spectrum confirming the presence of Sn and O, with the inset showing the corresponding elemental mapping and spatial distribution of Sn and O.

**Figure 6:**
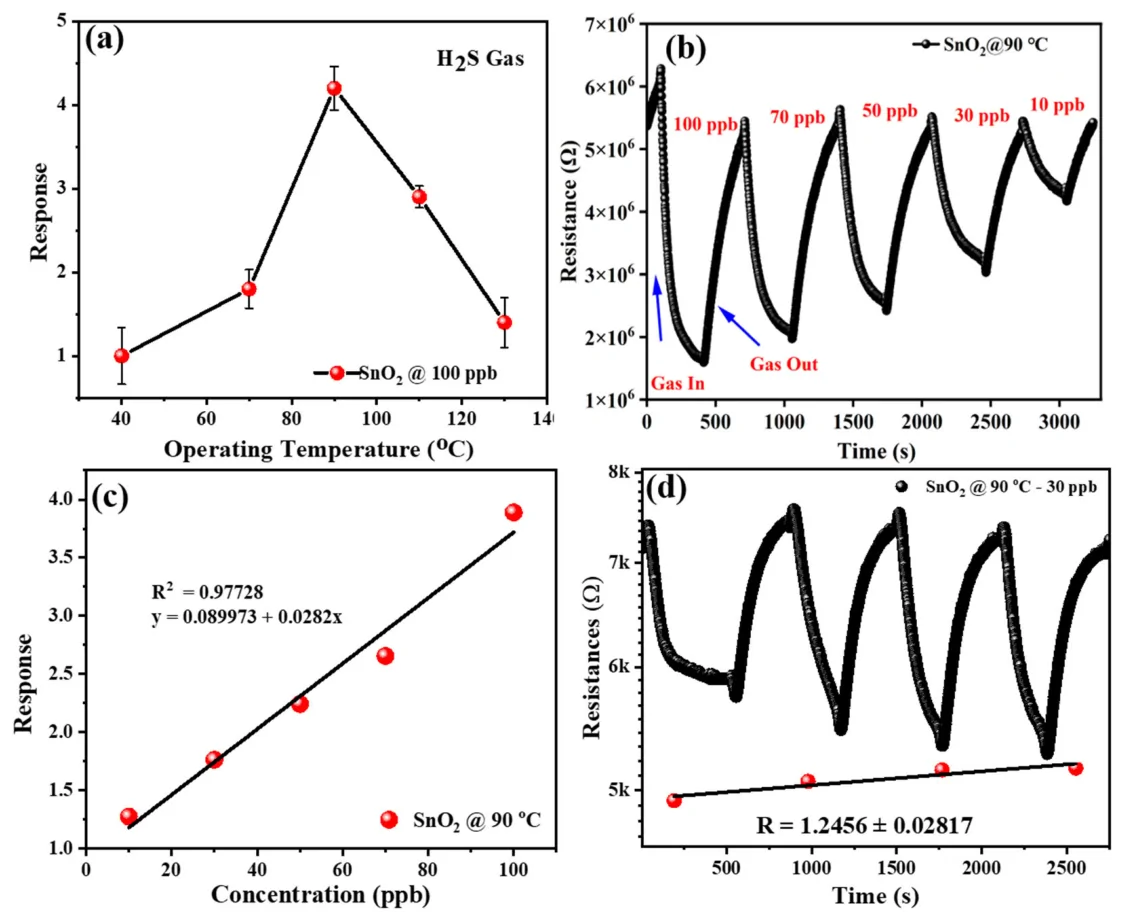
(**a**) Response of SnO_2_ sensor measured at different operating temperatures toward 100 ppb H_2_S gas; (**b**) dynamic response–recovery characteristics toward H_2_S concentrations ranging from 10 to 100 ppb at optimum operating temperature; (**c**) linear relationship of transient responses to concentrations of H_2_S; (**d**) repeatability of SnO_2_ sensor to 30 ppb H_2_S gas at 90 °C.

**Figure 7:**
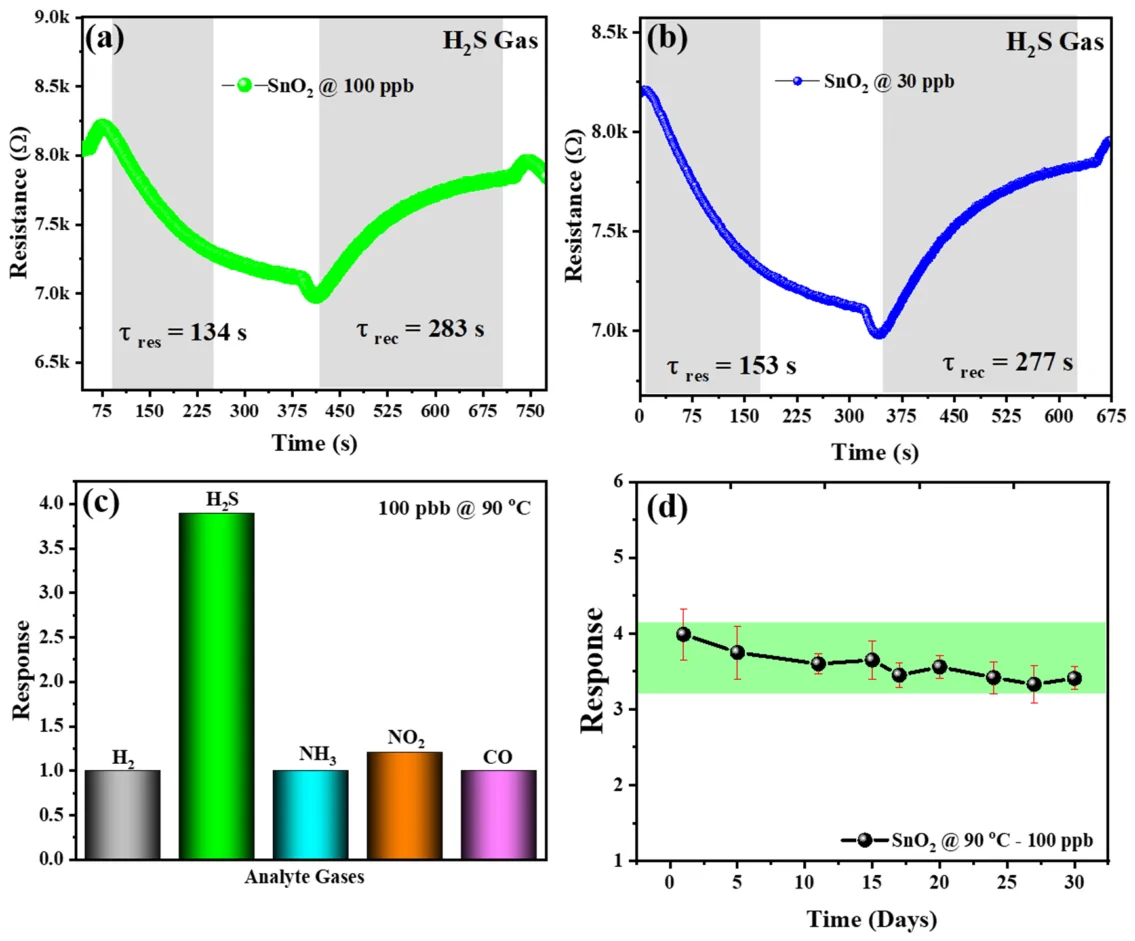
Response and recovery times of SnO_2_: (**a**) at 100 ppb; (**b**) at 30 ppb. (**c**) Selectivity of SnO_2_ sensor toward other interfering gases; (**d**) Stability over period of 30 days of SnO_2_ sensor at 90 °C (The green shaded region serves as a visual reference to indicate the range within which the response is considered sufficiently stable).

**Figure 8:**
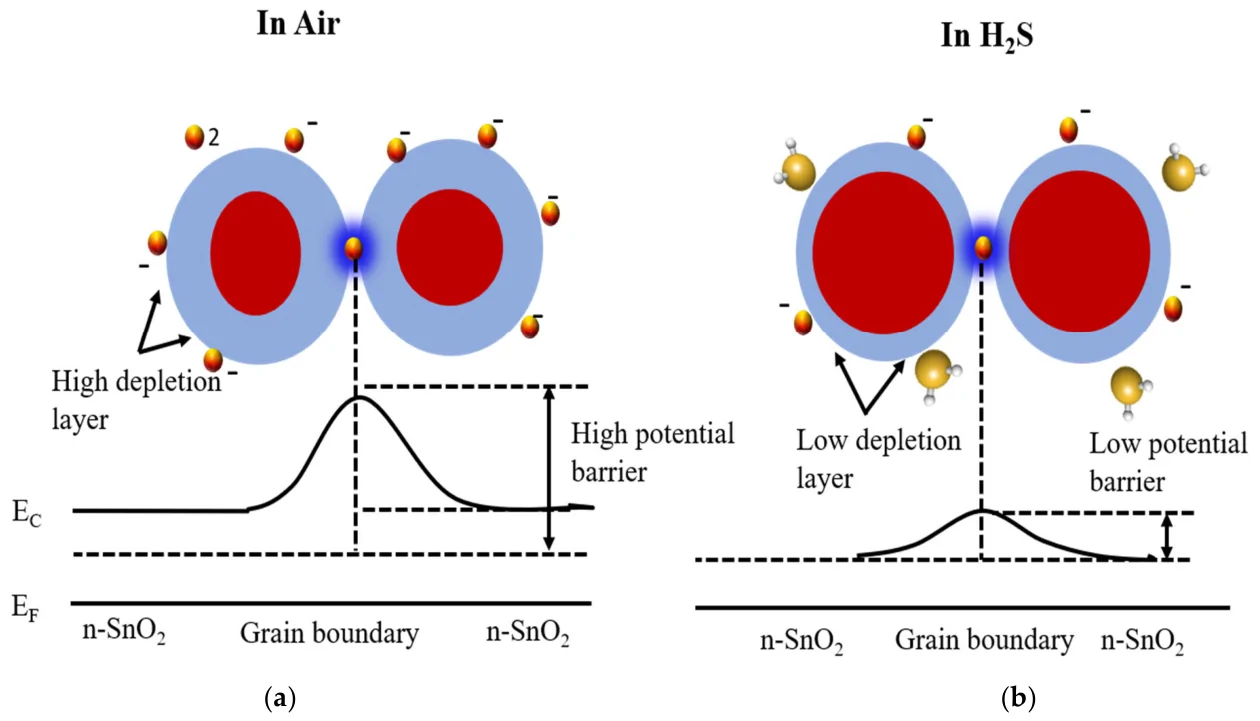
(**a**) Schematic illustration of possible sensing mechanisms of SnO_2_ MEMS sensor toward H_2_S gas; (**b**) Energy band diagrams of SnO_2_.

**Table 1:** Comparison of key performance parameters of proposed SnO_2_-based H_2_S sensor with representative previously reported sensors.

Materials	Deposition Method	T °C	PC (mW)	T_res_/T_rec_ s	Conc. (ppm)	Response	LOD (ppm)	References
TiO_2_/SnO_2_	Drop coating	300	100	25/173	5	5.9	1.8	[53]
ZnO/Co_12_	Drop coating	115	-	19/20	0.01	0.1	0.01	[54]
In/SnO_2_	Self-assembly	280	17.2	2.4/54.6	100	594	0.01	[55]
Ni/CeO_2_	Drop coating	200	20	8/13	0.5	78	0.01	[56]
Cuo/zno	Inkjet printed	230	-	-	20	16	0.0086	[57]
SnO_2_ NPs	3D dispensing printing	90	32	134/283	10	3.8	0.0035	This work

## Data Availability

The data presented in this study are available on request from the corresponding author. The original contributions presented in this study are included in the article. Further inquiries can be directed to the corresponding author.
